# The Bees and the Hive

**Published:** 1888-03-24

**Authors:** 


### The Doctor's Pulpit.

### THE BEES AND THE HIVE.

No argument is needed to convince anyone who has reached the age of manhood that the miscellaneous collection of bees which occupies the British hive is, in American parlance, "very considerably crowded." The hive is not very large, and the meadows and flower gardens from which honey may be gathered are by no means unlimited. A doctor, if disposed to be cynical, might find a certain scientific satisfaction in the crowding together of vast numbers of human beings in a small area. There is the possibility of studying the genus *homo* under every imaginable variety of circumstance. He can be seen stalled like a prize bullock, with every luxury in the way of eating, drinking, clothing, and pleasure which money can buy. Or he can be examined in a state of what may be called "civilised nature," that is to say, worse off in every respect than the most degraded savage of Australia or Africa. For the savage has at least the open sky above his head and a pure atmosphere about him; and if he chooses to devote himself for three hours a day to hunting or the cultivation of his little patch of ground, he can soon make himself a rich man—for a savage. But the man who is reduced to the lowest level in England, and especially in London, is in a very different condition. Does he ever get as much good food as he could enjoy, even though he works from sunrise to sunset all the year round? Does he ever see a perfectly clear sky, or know what it is to breathe a pure air?

Between the extremely rich and the extremely poor every variety of man and woman will be found. The coldly philosophical doctor, who is minded only to increase his knowledge and to evolve a sounding generalisation, such as the "struggle for existence," the "survival of the fittest," or other grammatical equivalent for facts which include such things as heartbreaks in their all-embracing range, learns thus to understand one aspect of human life, and takes a pride, not unmixed with pleasure, in the increase of his knowledge and the aptness of his theories and demonstrations. That is held to be one of the principal functions of the highly scientific man of physic. He sees, he records, he reasons, and he receives the applause of his fellows for his clear and critical expositions.

All these observations and generalisations about men in the mass—men in a state of high civilisation—are extremely valuable so far as they go. They at least make us see clearly what we have to deal with; and the first condition of successful work is to know exactly what kind of work it is you want to do. But now that we know exactly the state our towns are in; now that we see how many thousands of men and women are being ground to most miserable powder, by the sweating system, for example; now that we are convinced, and more than convinced, that there are twenty wolves for every single lamb, and that, however carefully the lamb is divided, some of the wolves get three or four shares each, whilst others get only the remains of the bones; now that we know all these things so well that no eloquence of tongue or pen could by any possibility make us know them better—what next?

What have the doctors—those of political economy and those of physic—to say to us at this stage of development? For we are ready to admit that the kind of doctor we admire most is not he who can most learnedly demonstrate the pathological process which is taking place in a given part of the patient's organism, nor he who knows most accurately the physiological actions of drugs in every conceivable dose and under every imaginable circumstance. Such an one may be, and probably is, a very clever man; but we confess to an irremovable prejudice in favour of the doctor who "believes in treatment." For really, what is the use of your physiology, your pathology, your pharmacology, and your "dismal science" of political economy, if nobody is to be the better for it?

It is precisely thus with the question of the overcrowding bees in the human hive:—they are stinging one another to death by hundreds every day. What is Dr. Beemaster going to do under the circumstances? Well, he can adopt one of two course. He can let them fight on, and take out the dead bees as fast as they die, and bury them; or he can make another hive and put it into another flower garden.

It is not after all so very difficult to know what *ought* to be done. The real question is, What has become of Dr. Beemaster? Nobody seems to know where he is. The Government says he is not at Whitehall. The House of Lords cannot find him in the gilded chamber. The Commons entirely deny any knowledge of him. Where is he and what is he doing?

In the absence of the Beemaster let the appeal be made to the bees themselves. Why should we stick to the parent hive so tenaciously when it is so terribly small? and why should we so perseveringly fly round and round the paternal gardens and meadows when almost all the honey has been long since sucked by our industrious predecessors, and there is hardly anything but dregs left? Are there no hives and no meadows elsewhere? That is a practical question, and demands a practical answer. There are plenty of spreading heathers in full bloom awaiting any adventurous bees who have the courage and the strength of wing to seek them.

This very week the writer received a letter from a foreign clime, where the land is flowing with milk and honey, where all kinds of fruits grow without cultivation, and all kinds of crops with a mere scratching of the surface of the ground. Close to the correspondent's home are hundreds of square miles of land fitted for every purpose of agriculture. It is true that this land of Goshen is in East Africa. It is true that here and there is a native village with its fickle-tempered chief. It is also true that occasional bands of Arabs prowl about in search of natives whom they may catch and sell for slaves. But what then? A thirteen years' resident states that a hundred Englishmen could hold their own against all the Arab robbers, and they could at the same time secure for themselves the eternal gratitude of the natives by protecting them against the horrors of slavery. A hundred Englishmen, well disciplined and led, could free a large district from one of the most terrible of curses, and gain at a stroke homes and plenty for thousands of their fellow countrymen, whose struggles in this country only end in mutual destruction.

There are certain people who are always talking about the spread of our Empire, and the increase of our responsibilities. We *must* spread or starve; and we are fools to starve so long as there is rich and unoccupied land in any part of the globe. If such spreading increases our responsibilities we must submit to the increase. It is imbecile to let theoretical objections keep us at home when going abroad would save us from practical starvation Many an Englishman never knows what excellent stuff there is in him until he is taken away from the props and buttresses and swaddling clothes of our old-world civilisation. When he finds himself put down in a new country where a home may be had for the conquering, and bread for the growing, but where idleness has no poor-law to appeal to, if there is an atom of manhood in his soul he soon shows that all he really wanted was a chance. That chance may be found in at least one part of the world. Those bees who find their own hive too full, and the flowers of their meadows destitute of honey, should swarm off to fresh fields and happier skies elsewhere. This is what men of all generations in all overcrowded countries have had to do; and this is what we must do, or die in our nests like owls who starve because they have not the courage to seek mice a few miles from home.

